# Population epigenetic divergence exceeds genetic divergence in the Eastern oyster *Crassostrea virginica* in the Northern Gulf of Mexico

**DOI:** 10.1111/eva.12912

**Published:** 2020-01-07

**Authors:** Kevin M. Johnson, Morgan W. Kelly

**Affiliations:** ^1^ Department of Biological Sciences Louisiana State University Baton Rouge LA USA

**Keywords:** *Crassostrea virginica*, DNA methylation, epigenetics, oyster, reduced representation bisulfite sequencing, salinity

## Abstract

Populations may respond to environmental heterogeneity via evolutionary divergence or phenotypic plasticity. While evolutionary divergence occurs through DNA sequence differences among populations, plastic divergence among populations may be generated by changes in the epigenome. Here, we present the results of a genome‐wide comparison of DNA methylation patterns and genetic structure among four populations of Eastern oyster (*Crassostrea virginica*) in the northern Gulf of Mexico. We used a combination of restriction site‐associated DNA sequencing (RADseq) and reduced representation bisulfite sequencing (RRBS) to explore population structure, gene‐wide averages of *F*_ST_, and DNA methylation differences between oysters inhabiting four estuaries with unique salinity profiles. This approach identified significant population structure despite a moderately low *F*_ST_ (0.02) across the freshwater boundary of the Mississippi river, a finding that may reflect recent efforts to restore oyster stock populations. Divergence between populations in CpG methylation was greater than for divergence in *F*_ST_, likely reflecting environmental effects on DNA methylation patterns. Assessment of CpG methylation patterns across all populations identified that only 26% of methylated DNA was intergenic; and, only 17% of all differentially methylated regions (DMRs) were within these same regions. DMRs within gene bodies between sites were associated with genes known to be involved in DNA damage repair, ion transport, and reproductive timing. Finally, when assessing the correlation between genomic variation and DNA methylation between these populations, we observed population‐specific DNA methylation profiles that were not directly associated with single nucleotide polymorphisms or broader gene‐body mean *F*_ST_ trends. Our results suggest that *C. virginica* may use DNA methylation to generate environmentally responsive plastic phenotypes and that there is more divergence in methylation than divergence in allele frequencies.

## 1 INTRODUCTION

In highly variable marine environments, phenotypic plasticity allows organisms to persist in suboptimum environmental conditions (Somero, 2010). On short time scales, this can be accomplished via environmentally responsive changes in gene expression (Evans & Hofmann, 2012). Over the medium to long term, there is increasing evidence that phenotypic plasticity may be modulated by changes in the epigenome (Eirin‐Lopez & Putnam, 2018; Hofmann, 2017; Hu & Barrett, 2017; Verhoeven, vonHoldt, & Sork, 2016). These epigenomic changes allow for the persistent expression of a plastic phenotype that in some cases may be transmitted to subsequent generations (Rondon et al., 2017). As a result, there is significant interest in understanding how epigenetic variation correlates with genomic and environmental variation; and ultimately, how changes in the epigenome might facilitate the generation of environmentally responsive phenotypes. The push to address these questions has developed as a result of new sequencing techniques that provide high‐resolution data on epigenomic traits and the necessary data for identifying when phenotypic effects of genotype‐by‐environment interactions are governed by changes in the epigenome (Trucchi et al., 2016; Van Gurp et al., 2016; Yaish, Peng, & Rothstein, 2014).

While there are multiple types of epigenetic mechanisms, 5mC DNA methylation (methylation at the 5th position in a cytosine molecule) has been the focus of a substantial amount of research to date using enzyme‐linked immunoglobulin assays, methylation sensitive PCRs, and modified amplification polymorphism assays (Hu & Barrett, 2017; Kilvitis et al., 2014; Metzger & Schulte, 2016; Schrey et al., 2013). These methodologies are able to identify quantitative changes in DNA methylation; however, more targeted hypothesis testing is limited by a lack of high‐resolution nucleotide‐specific data among nonmodel marine invertebrates (Hofmann, 2017; Schrey et al., 2013). By contrast, reduced representation bisulfite sequencing (RRBS) methods provide nucleotide specific 5mC DNA methylation status using high‐throughput short‐read sequences on the Illumina platform (Trucchi et al., 2016; Van Gurp et al., 2016). In particular, the epiGBS (Van Gurp et al., 2016) method combines this RRBS approach with enzyme digestion that is not sensitive to methylation and therefore provides locus‐specific methylation state across a large proportion of the genome, even when no reference genome is available.

Much of what is known about the functional role of epigenetic modifications in sessile marine invertebrates comes from research on the Pacific oyster (*Crassostrea gigas*). In *C. gigas*, DNA methylation appears to be an important molecular feature that directs developmental plasticity, gene regulation, and control over alternative splicing (Olson & Roberts, 2014; Riviere, 2014; Riviere et al., 2013; Song, Li, & Zhang, 2017). Specifically, increased levels of DNA methylation within intragenic regions in *C. gigas are* positively correlated with gene expression levels (Gavery & Roberts, 2013), while exon methylation is positively correlated with inclusion in mRNA transcripts (Song et al., 2017). Furthermore, changes in methylation patterns following parental exposure to environmental toxins appear to be heritable in the F1 generation (Rondon et al., 2017). Methylation research in *C. gigas* has been facilitated by the availability of a reference genome (Trucchi et al., 2016). The recent release of a reference genome for the Eastern oyster *Crassostrea virginica* (Gómez‐Chiarri, 2018) provides an opportunity to explore similar patterns of DNA methylation with RRBS, and opens the door to future comparative epigenomic studies between these two closely related taxa.

Previous studies testing for acclimatization responses to divergent environments have focused on comparative transcriptomics to measure differences in global gene expression among populations across these conditions (DeBiasse & Kelly, 2016; Stillman & Armstrong, 2015). These differences may themselves be modulated by genome‐level epigenetic changes, which may also impart this information to the next generation (intergenerational plasticity) (Hofmann, 2017; Platt, Gugger, Pellegrini, & Sork, 2015; Vu, Chang, Moriuchi, & Friesen, 2015; Wong, Johnson, Kelly, & Hofmann, 2018). The regulatory importance of epigenetic changes in the process of population divergence is increasingly being supported by models that suggest these nongenetic features influence the rate of adaptation to environmental stress (Klironomos, Berg, & Collins, 2016; Kronholm & Collins, 2016). Therefore, to better understand the genetic basis for population divergence, the interacting effects of genetic and epigenetic variation should be further explored.

Recent studies in wild populations have identified variation in DNA methylation that is associated with both acclimatization and broader population‐specific epigenomic variation. These studies have identified methylation differences between freshwater and marine populations of the three‐spined stickleback (*Gasterosteus aculeatus*) (Artemov et al., 2017) and between invasive and native populations of the pygmy mussel (*Xenostrobus secures*) (Ardura, Zaiko, Morán, Planes, & Garcia‐Vazquez, 2017). Furthermore, multiple comparisons in species from clonal fish, tilapia, bats, and finches have found variation in DNA methylation that is greater than the standing genetic variation, lending support for the role of DNA methylation in alleviating the consequences of genotype‐by‐environment mismatches in a wide range of metazoans (Liu, Sun, Jiang, & Feng, 2015; Massicotte, Whitelaw, & Angers, 2011; Skinner et al., 2014; Wan et al., 2016).

Genotype‐by‐environment interactions shape population structure and can play an important role in establishing population specific DNA methylation patterns and in driving phenotypic differences between populations. Recent evidence has shown that reduced representation genomic sequencing methods such as restriction site‐associated DNA sequencing (RADseq) are a valuable tool for evaluating population structure in marine invertebrates (Bernatchez et al., 2019; Silliman, 2019; Xuereb et al., 2018). The population differences that have been detected in many of these studies are surprising, as many marine invertebrates are broadcast spawners with large dispersal distances, a feature that should produce high gene flow across a species’ range (Sanford & Kelly, 2011). However, previous assessments of population structure along the northern Gulf of Mexico (GOM) in Eastern oysters (*Crassostrea virginica*) have identified genetic variation between populations (Anderson, Karel, Mace, Bartram, & Hare, 2014). These genetic differences may be explained by either a shift in phenology between regions of the GOM or by the influence of genotype‐by‐environment mismatches removing some genotypes from any given estuary. In the northern GOM, one potential driver of genotype‐environment mismatches is variation in salinity (Leonhardt, Casas, Supan, & Peyre, 2017).

In coastal Louisiana, populations of *Crassostrea virginica* are spread across estuaries that range from low salinity (<5 psu) to mid‐high salinity (~20 psu) (Das et al., 2012). Within these estuaries, *C. virginica* plays a critical role as both a foundation species and an ecosystem engineer (Meyer, Townsend, & Thayer, 1997; Plunket & La Peyre, 2005). Previous research has shown that along the northern GOM, the interaction between temperature and salinity plays a major role in setting the species’ environmental distribution (Eastern Oyster Biological Review Team, 2007; Shumway, 1996). While broad scale genotyping of oyster populations along the GOM has provided some evidence for genetic differentiation, there is little known about whether this genetic variation contributes to local adaptation to salinity (Varney, Galindo‐Sánchez, Cruz, & Gaffney, 2009). In addition, regional salinity levels are also predicted to change dramatically in estuarine ecosystems due to changes in rainfall and anthropogenic alterations to hydrology (Das et al., 2012; Powell & Keim, 2015). Previous research into the molecular responses of *Crassostrea spp.* to environmental variation has identified significant responses to salinity at both the mRNA and protein levels (Chapman et al., 2011; Jones, Johnson, & Kelly, 2019). Therefore, examining the degree of genetic and epigenetic variation between populations of *C. virginica* that inhabit estuaries with distinct salinity conditions is an important step in understanding the observed signals of local adaptation and phenotypic plasticity in response to changes in environmental conditions along the northern GOM.

**Figure 1:**
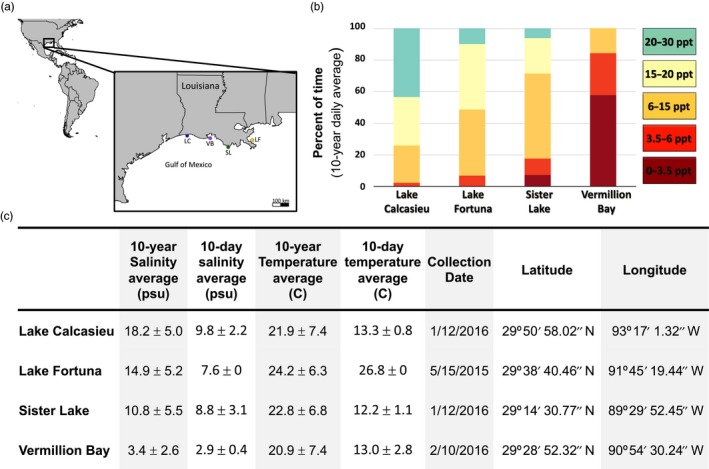
Site‐specific variation in salinity. (a) Map indicating site locations along coastal Louisiana. (b) 10 year mean distribution of time spent at each salinity bin. (c) Table of mean salinity and temperature for each site along with data of sample collection and coordinates of oyster reef

In this study, we characterize single nucleotide polymorphisms and DNA methylation patterns for four populations of Eastern oysters (*Crassostrea virginica*) along the northern GOM. This region is highly variable with respect to salinity, with shallow oyster reefs existing across regions with distinct annual salinity profiles (Figure 1). Recent evidence suggests population‐specific physiological responses to salinity between oysters from either high or low salinity reefs from this region (Leonhardt et al., 2017). In addition, we hypothesized that the Lake Fortuna population, located east of the Mississippi river, would be the most unique population both in terms of methylation and genomic variation. As such, we have used a combination of RADseq and epiGBS sequencing to further describe potential genetic and epigenetic drivers of these observed population‐specific physiological responses.

## 2 MATERIALS AND METHODS

### 2.1 Collection sites

Oysters were collected from four sites that are known to differ in annual mean salinity. Salinities for each estuary were calculated as daily averages from June 1, 2006, to June 1, 2016, using salinity measurements (collected by either the United States Geological Survey or the Louisiana Department of Fish and Wildlife) from stations located as close to the oyster beds as possible (Figure 1). These data were used to calculate the amount of time each reef spent at one of five salinity regimes known to impact oysters in the eastern GOM (which has higher average salinities than the northern GOM). These bins were (a) >20 practical salinity units (psu)—no negative effects, (b) 15–20 psu—reduced reproductive rates, (c) 6–15 psu—reduced spat recruitment, (d) 3.5–6 psu—reduced growth rates, and (e) 0–3.5 psu—survival limited to approximately 5 months (Barnes, Volety, Chartier, Mazzotti, & Pearlstine, 2007). In addition, average salinity and temperature for the 10 days prior to collection was also calculated and reflects the environmental conditions prior to sample collection.

### 2.2 Oyster collections

In order to characterize population‐specific methylome patterns, 20 individual oysters from each site; Lake Calcasieu (LC), Lake Fortuna (LF), Sister Lake (SL), and Vermillion Bay (VB);were collected as part of a larger study between May 15, 2015, and February 10, 2016 (Figure 1). Oyster from all sites was collected by dredging at a depth of ~3 m allowing for all oysters to have been collected from subtidal reefs. Salinity and temperature data were collected, and 10‐year daily average is reported in addition to the average conditions for the 10 days preceding collection (Figure 1c). Following collections, oysters were transported to Louisiana State University; gill tissue was dissected from each oyster, immersed in 95% ethanol, and stored at +4°C for 18–24 months. The remaining whole organisms were subsequently frozen and stored at −80°C. In order to gather morphological data, these oysters were thawed in September 2019. For individuals from LF and SL, individual specific length, wet weight, and presence/absence of gonads was assessed; however, due to freeze‐thaw, it was not possible to identify the current sex of the individual. As oysters are sequential, protandric hermaphrodites, sexual stage has to be checked via gonads (Harding, Powell, Mann, & Southworth, 2013). For the other two sites (LC and VB), all 20 individuals were measured and weighed; however, the labeling of these individuals was lost, and so, only mean length and mean wet weight are reported.

### 2.3 DNA extraction

DNA was extracted from all 80 individuals using the OMEGA E.Z.N.A. Tissue DNA Kit (D3396‐01; Omega bio‐tek) with a 2 min RNase A digestion to remove co‐purified RNA. Extracted DNA purity was assessed on 260/280 and 260/230 ratios using a nanodrop spectrophotometer (ND1000; Thermo fisher Scientific). Presence of high molecular weight DNA was confirmed using a 1.5% agarose gel, and DNA concentration was verified using a Qubit 3.0 Flourometric dsDNA BR assay kit (Q32850; Life Technologies).

### 2.4 RADseq library preparation

RADseq libraries were generated for the same 80 individuals plus an additional two individuals from each population providing (*n* = 88) using the 3RAD library preparation method (Bayona‐Vásquez et al., 2019). For these libraries, 100 ng of DNA was digested in a 15 µl reaction consisting of three enzymes (XbaI, EcoRI, and NheI, New England BioLabs (NEB)), an iTru NheI adapter, an iTru EcoRI adapter, and NEB 10× CutSmart Buffer. Reactions were incubated for 2 hr at 37°C. The adapters added in the original reaction were subsequently ligated to the digested DNA fragments with the addition of one unit of DNA ligase, 10× ligase buffer, additional rATP to ensure ligation of the iTru adapters, and the sample incubated at 22°C for 20 min. Ligation products were pooled, purified with 1.25× speedbeads, and amplified using 1 unit of Kapa HiFi DNA Polymerase (KapaBiosystems) in the presence of unique i5 and i7 primers. Amplification was completed using 12 cycles of 98°C for 1 min; then, 12 cycles of 98°C for 20 s., 60°C for 15 s, 72°C for 30 s; and 72°C for 5 min. Final library concentrations were quantified via qPCR, and sequencing was completed on 1 lane of an Illumina Hiseq2500 with 100bp paired‐end reads. Sequencing was completed at the North Carolina State University's Genomic Sciences Laboratory (Raleigh, NC).

### 2.5 RADseq data analysis

The 3RAD data for the 88 individuals were demultiplexed using the process_radtag command available in STACKS v.1.48 (Catchen, Hohenlohe, Bassham, Amores & Cresko, 2013) where sequences with any uncalled base were removed (−c), low quality reads removed (−q), reads trimmed to 140 bp (−t 140), RAD‐Tags rescued (−r), and a score limit of 10 (−s 10). The program Bowtie2 v.2.3.4 (Langmead & Salzberg, 2012) was then used for alignments to the reference genome (GCF_002022765.2) with default settings. The STACKS (Catchen et al., 2013) software was used to isolate SNPs between the four populations. The stack analysis was run using the *ref_map.pl* pipeline with the following settings “‐p 2 ‐‐smooth ‐‐hwe ‐r 0.65 ‐‐min_maf 0.05 ‐‐bootstrap ‐‐bootstrap‐reps 1000000 ‐‐structure ‐‐genepop ‐‐write_single_snp –vcf.” These settings requires a locus to be represented by 65% of individuals in two of the four populations that have a minimum allele frequency of 0.05, *F*_ST_ values are kernel‐smoothed across chromosomes, and significance intervals based on resampling using 1,000,000 bootstraps (Appendix S1).

**Figure 2:**
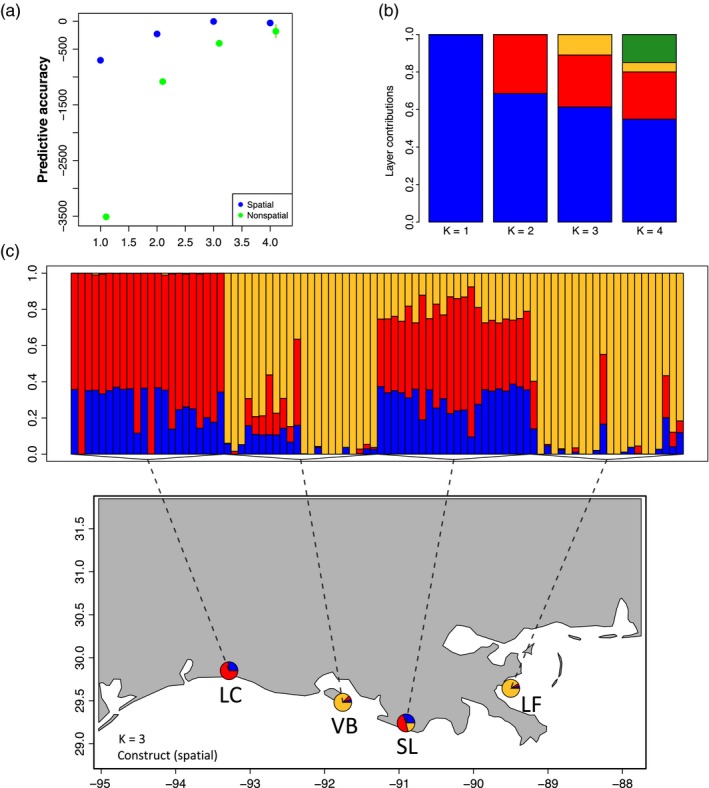
(a) ConStruct cross‐validation analysis predictive accuracy of *K* from 1 to 4 using 90% of the dataset for training, 10 replicates, and 10,000 iterations per replicate. (b) ConStruct layer contributions of *K* from 1 to 4. (c) Population structure plot of *K *= 3 mapped to estuary locations. Downstream analysis of methylation focused on a *K *= 2 model that groups LC with SL and VB with LF

Population structure was investigated using the R program conStruct (v.1.0.3) and the population structure file generated from the Stacks program *population.* Samples were analyzed using the *x.validation* function in conStruct testing *K* from 1 to 4 using 90% of the dataset for training, 10 replicates, and 1,000 iterations per replicate (Bradburd, Coop, & Ralph, 2018). Input coordinates for each population are the same as those presented in Figure 1c. The final *K* was chosen based on comparing layer contributions and predictive accuracy for models constructed both with and without geographic distances between each of the four estuaries (Figure 2a,b).

### 2.6 DNA methylation epiGBS library preparation

For epiGBS library preparation, a total of 500 ng of purified genomic DNA was simultaneously double digested for all 80 samples in a 96‐well plate using the two frequent cutter enzymes AseI and NsiI (NEB‐R0127L and NEB‐R0526L; Van Gurp et al., 2016). Digested DNA was ligated to custom y‐yoked methylated sequencing adapters (Glenn et al., 2016) using a T4 DNA ligase (B9000S; New England Biolabs) with additional rATP to ensure ligation of custom adapters (Appendix S1). Digested and methylated adapter‐ligated DNA was bisulfite converted in a 96‐well plate using the Zymo Research EZ DNA Methylation‐Lightning kit (D5031; Zymo Research) with a 15 min L‐desulphonation step. Bisulfite converted DNA was then tagged and amplified with Illumina adapters using 16 cycles of PCR. Amplified libraries were size selected to 300–600 base pairs (bp) using the Zymo Research Select‐A‐Size DNA clean & Concentrator (D4080; Zymo Research). Size selection was confirmed using the Agilent Bioanalyzer DNA high sensitivity chip (5067–4626; Agilent Technologies). Libraries were subsequently pooled and sequenced with a 10% PhiX spike‐in on a full flow cell of the Illumina NextSeq 500 with 75 bp paired‐end reads. Sequencing was completed at the North Carolina State University's Genomic Sciences Laboratory (Raleigh, NC).

### 2.7 Differential DNA methylation analysis

The epiGBS sequencing reads were adapter and quality trimmed using *Trim Galore!* (v.0.4.5) (Krueger, 2015) with default settings. Trimmed, nondirectional, paired end reads were mapped to the reference genome, and CpG methylation was called using the software package *bismark* (v0.19.0) (Krueger & Andrews, 2011). The *bismark* commands used in the mapping allowed for 1 mismatch in a seed alignment of 10 with a minimum alignment score setting of −0.6 (‐‐score_min L, 0, −0.6). These settings were selected to account for genomic variations between *C. virginica* collected from the northern GOM (this study) and the disease‐resistant inbred line from the U.S. East Coast used for the construction of the reference genome (Gómez‐Chiarri, Warren, Guo, & Proestou, 2015). CpG methylation was extracted from the nondeduplicated mapped reads using the *bismark* command *bismark_methylation_extractor* with the following commands; ‐‐ignore_r2 2, ‐‐bedGraph, ‐‐zero_based, ‐‐no_overlap, ‐‐cytosine_report, and –report. Differential methylation was conducted on CpG features using the bismark coverage files and the R program *MethylKit* (v.1.2.4) (Akalin et al., 2012). Loci were first filtered using the *filterByCoverage* command to require a locus to have a low count of at least 10 reads to be included.

Methylation was then assessed in a pairwise bases using a 100 bp tiled approach with a step size of 100 bp and restricted to tiles that had been covered by at least eight individuals from each of the two populations. These parameters were chosen so that each 100‐bp region would contain at least 1 CpG, would be approximately be equivalent to a single sequencing read (75 bp), and would be present in 40% of the population. Pairwise assessments of differential methylation were focused on contrasting the high salinity population with the other three populations. As such, the analysis described here reflects on both abiotic differences between estuaries over the 10‐year daily mean differences between sites and the 10‐day daily mean differences prior to sampling (Figure 1c). Significantly differentially methylated regions (DMRs) were identified for all comparisons using a minimum percent methylation difference between populations of 15% and a Benjamini‐Hochberg adjusted *p*‐value (*q*‐value) less than or equal to .05 (Mathers et al., 2019).

All 100‐bp regions with sufficient coverage in at least one of the pairwise comparisons were imported into *SeqMonk* (v1.40.1; http://www.bioinformatics.babraham.ac.uk/projects/seqmonk/) for visualization and annotation with nearest gene annotation. Each of these 100‐bp regions were mapped to the nearest genomic feature *SeqMonk* and compared with the randomly generated sequencing markers. The genomic features included in this analysis were promoter regions (2 kb upstream of gene), 3′UTRs, introns, exons, 5′UTRs, downstream regionus (within 2 kb downstream of gene), and intergenic regions. To investigate potential for the identified DMRs to be a result of random sampling, *SeqMonk* was used to generate 6 genome‐wide sets of 100 bp tiles (*n* = 39,053). The percent overlap of methylated genomic features for each marker was assessed using the R program *ChIPpeakAnno* (v 3.10.2) (Zhu et al., 2010) and the distribution of DMRs for each comparison tested for significant differences in distribution when compared with the random markers using the Kolmogorov–Smirnov test (*p*‐value ≤ .05).

Differentially methylated regions were annotated to the nearest gene body (defined here as the regions including promoters, introns, exons, 3′ and 5′ untranslated regions) using the sequence annotations available with the latest release of the *C. virginica* genome (GCF_002022765.2, version 3.0). This definition of gene body was used as these neighboring regions all have the capacity to modify gene expression and therefore directly affect phenotypes (Gavery & Roberts, 2013; Keller, Han, & Yi, 2016; Song et al., 2017). These genome annotations were combined with gene ontology terms generated in this study using *InterProscan 5* (v 5.27–66.0) and the most recent gene ontology database release (format‐version: 1.2, release: 2018–02–20). Gene ontology enrichment analysis was performed with R scripts for rank‐based gene ontology with adaptive clustering (github.com/z0on/GO_MWU) and was implemented with a signed log of *q*‐values as a continuous measure of significance (Wright, Aglyamova, Meyer, & Matz, 2016). Significantly enriched gene ontologies for each ontology (i.e., biological process, cellular components, and molecular functions) were called based on an adjusted *p*‐value less than .05. In all of these calculations of gene ontology enrichment, the background gene set was comprised of all genes that were identified within at least one population.

### 2.8 Associating between DNA methylation and genomic estimate of F_ST_

To investigate the overlap between genomic variation and changes in DNA methylation, we reduced both datasets so that for each gene in each pairwise comparison, there would be a single *F*_ST_ value that represented the weighted average *F*_ST_ observed across a gene, an average percentage of gene‐body methylation for each gene, and a measurement of epigenetic divergence (st). We defined *P*_ST_ as the methylation analogue of Wrights *F*_ST_ and calculated it for each locus by subtracting the total variance in methylation in all populations from the variance within a single population and divided by the variance in all populations (*P*_ST_ = (Variance_Total_ − Variance_Sub_)/Variance_Total_; Leinonen, McCairns, O’Hara, & Merilä, 2013). Population level variances in methylation were calculated from the bismark coverage output, and variance within each population was calculated using the rowVars command in the R stats package “matrixStats.” The distribution of mean *F*_ST_ among the DMRs for each comparison was tested for significant differences in distribution when compared with the mean *F*_ST_ for all region using the Kolmogorov–Smirnov test (*p*‐value ≤ .05).

Finally, to explore the influence of population structure, samples were grouped based on the results from an interpretation of the conStruct analysis contrasting individuals based on *K* = 2 with a combined pool of Lake Fortuna and Vermilion Bay from the combined samples of Sister Lake and Lake Calcasieu (Figure 2). These methylation data were analyzed by requiring a tile to be represented by at least 8 individuals per population and differential methylation assessed based on a minimum percent methylation difference of 15% and a *q*‐value less than or equal to .05. Hierarchical clustering for both the four population and two population samples was also completed using the methylKit function *clusterSamples* that applies Ward's hierarchical clustering using 1‐Pearson's correlation distances (Akalin et al., 2012).

## 3 RESULTS

### 3.1 Salinity variation among sites

Long‐term salinity data for each site confirm variation in mean salinity, with Lake Calcasieu (LC) > Lake Fortuna (LF) > Sister lake (SL) > Vermillion Bay (VB; Figure 1). Examining distribution of time spent within each of the five salinity stress indices underscores the increasing frequency of time spent in low salinity conditions for VB and SL as compared with the two mid‐high and high salinity sites (Figure 1). Specifically, LC and LF have recorded salinities greater than 15 psu for over 50% of the year, while SL is below 15 psu for 71% of the year, and VB is below 3.5 for 57% of the year. These differences in exposure history support the use of these oyster reefs in our efforts to describe population‐specific methylation profiles that may facilitate population differentiation and possibly local adaptation to low and high salinity in this region.

### 3.2 RADseq sequencing results

Sequencing of the 88 pooled RADseq libraries resulted in 232.8 (±221) thousand reads per library, with 97.5% of sequences retained after filtering. About 73,128 loci of which 3,169 passed population coverage filtering comprised of 1,134,111 nucleotides. Of these loci, 1,567 variant loci were retained for downstream analysis. Pairwise population *F*_ST_ values were calculated for the 1,567 polymorphic sites passing filter, and mean *F*_ST_ across all populations was found to be 0.023. Population structure modeling using the conStruct model that incorporated geographic distance identified *K* = 3 as significant; however, this analysis only identified slightly more support for a *K* = 3 over *K* = 2 (Figure 2a,b). We interpret these results to suggest that while *K* = 3 is the best fit for the data, a *K* = 2 explains close to the same amount of population structure such that Lake Calcasieu and Sister Lake are highly similar and Lake Fortuna and Vermilion Bay are also highly similar populations (Figure 2).

### 3.3 Sequencing statistics

**Figure 3:**
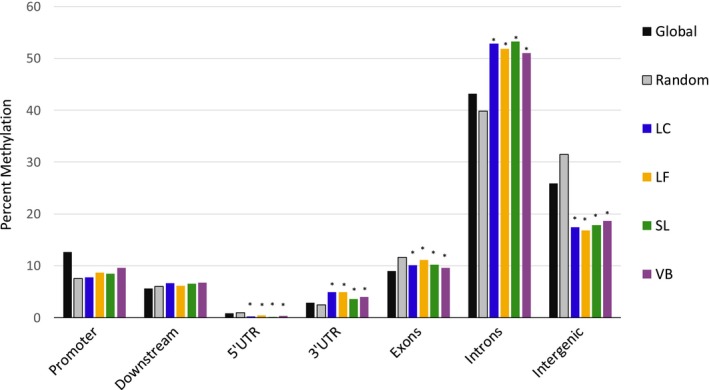
Distribution of percent methylation across genomic regions. Global DNA methylation (black) and the mean distribution of randomly generated markers (gray) distribution of methylated cytosines across gene regions. Site specific mean methylation of DMRs for each genomic region is also shown in color; (Blue—Lake Calcasieu; Orange—Lake Fortuna; Green—Sister Lake; Purple—Vermillion Bay). Stars indicate samples are significantly different from the mean distribution of random markers

Sequencing of the 80 pooled epiGBS libraries resulted in 1.3 (±0.79) million reads per library, with 80.2% of all sequences mapping to the reference genome (range: 74.6%–93.4%). There were 8,395,168 cytosines per sample sequenced at least once with 13.9% of genomic cytosines identified as methylated from the Bismark mapping results. Tiled differential methylation analysis identified a total of 3,643–100 bp regions covered by at least 8 individuals with 10× coverage within at least one of the four populations (*q*‐value ≤ .05). The distribution of these methylated regions revealed that they were predominately located within gene bodies (55.7%; 5’UTR, introns, exons, and 3’UTRs) and intergenic regions (25.9%), while 12.7% and 5.7% of methylated features were associated with gene promoter and downstream regions, respectively (Figure 3).

### 3.4 Differential methylation

**Figure 4:**
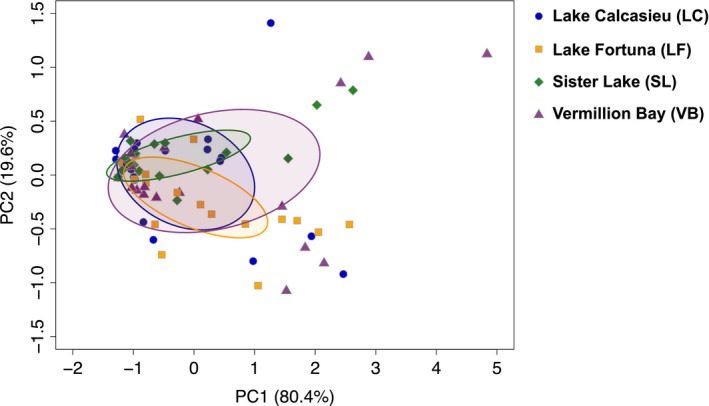
Principle component analysis plot of PC1 and PC2 of the global variation in methylation between populations (based on 900 shared regions). Ellipses display the standard deviation within PC1 and PC2 for each group revealing significant overlap

The principle component analysis (PCA) revealed that the majority of samples between the four populations showed overlapping means (Figure 4). Overall, 75.8% of all differentially methylated regions from all of the pairwise comparisons were located within gene bodies (promoters, introns, exons, 3′ and 5′ untranslated regions), 6.5% were within downstream regions, and 17.7% were located along intergenic regions (Figure 3). These distributions were tested against the randomly generated 100 bp regions and showed that the distribution of DMRs was higher than expected for introns, 3′ UTRs, and 5′ UTRs (*p*‐value ≤ .012); lower than expected for exons and intergenic regions (*p*‐value ≤ .008); but not significantly different from random for promoter (2 kb upstream) or downstream (2 kb downstream) regions (*p*‐value > .05). Finally, gene ontology enrichment for each population‐specific differential methylation analysis was conducted for each pairwise comparison between DMRs that fell within the gene‐body region using a Mann–Whitney *U* test with a background list of all genes for which there were sequence data within any of the comparisons.

### 3.5 Site‐specific DNA methylation trends

**Figure 5:**
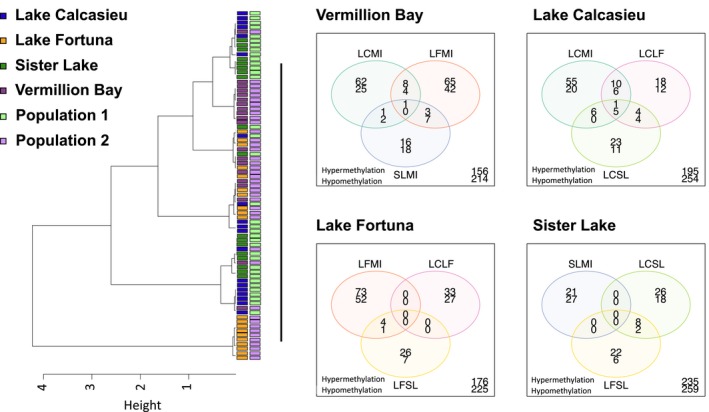
(a) Hierarchical clustering of CpG methylation between all four populations and the two population comparisons using Ward's hierarchical clustering with 1‐Pearson's correlation distances. (b–d) Venn diagrams revealing overlap of population‐specific differentially methylated regions that are driving hierarchical clustering observed in (a). Values presented represent significantly differentially methylated regions with hypermethylated regions printed on top and hypomethylated regions printed below

Oysters from the Lake Calcasieu (LC) site experience the highest annual and short‐term mean salinity conditions, spending approximately 74.2% of the year above 15 psu (Figure 1). Differential methylation for LC versus and each of the other three sites (LF, SL, and VB) identified 174 (8.0%) of the 100 bp methylated regions as significantly differentially methylated. Of these, only six DMRs were differentially methylated in all three population comparisons against LC. Comparing LC with the low salinity site, VB identified 103 DMRs while contrasts with the two mid‐salinity sites (LF and SL) contained 60 and 54 DMRs, respectively (Figure 5). Across all regions of gene bodies (2 kb upstream to 2 kb downstream), the most significant (lowest *q*‐value) hypomethylated DMRs in the LC population were associated with genes coding for an uncharacterized long noncoding RNA, a proton‐coupled folate transporter, and (in one comparison) the enzyme ketohexokinase‐like (Table 1). In addition, the most significant hypermethylated DMRs in the LC population were associated with genes coding for axonemal‐like dynein heavy chain 8 gene, an atrial natriuretic peptide receptor 1‐like isoform X3, and a serine/threonine‐protein phosphatase subunit (Appendix S2). Gene ontology (GO) enrichment for DMRs that fell within gene bodies revealed enrichment in two biological process ontologies between LC and SL oysters. These ontologies were *carboxylic acid metabolic process* and *cellular amino acid metabolic process*. GO enrichment also identified 2 molecular function ontologies between LC and VB which were identified as *guanyl‐nucleotide exchange factor activity* and *S‐adenosylmethionine‐dependent methyltransferase activity*.

**Table 1:** Significantly differentially methylated regions (DMRs) identified from the pairwise comparisons

Gene name	Gene ID	Chromosome	Start	LC versus SL methylation difference (%)	LC versus LF methylation difference (%)	LC versus VB methylation difference (%)
Uncharacterize lncRNA	gene3398	NC_035780.1	56,803,901	−21.98	−17.91	−15.66
Uncharacterized protein LOC111122494	gene10033	NC_035782.1	33,839,401	−15.19	−16.57	−18.26
TATA‐binding protein‐associated factor 172‐like	gene7372	NC_035781.1	50,910,301	NA	−26.67	−21.46
Proton‐coupled folate transporter‐like	gene16442	NC_035783.1	56,622,601	−21.57	−18.81	−15.11
LOW QUALITY PROTEIN: dynein heavy chain 8. axonemal‐like	gene10035	NC_035782.1	34,231,901	27.12	16.26	22.54
Atrial natriuretic peptide receptor 1‐like isoform X3	gene17788	NC_035784.1	16,257,601	15.09	17.94	15.70
Serine/threonine‐protein phosphatase 4 regulatory subunit 3A‐like	gene15310	NC_035783.1	37,214,601	NA	16.36	17.10
Ketohexokinase‐like	gene19389	NC_035784.1	38,858,801	NA	15.18	−27.35

Note

All comparisons with percent methylation difference had a *q*‐value < 0.05. Full list of DMRs is available in Appendix S2.

The Lake Fortuna site has the second highest annual mean salinity with approximately 51% of the year spent above 15 psu (Figure 1). This site also differed from all others in timing of the sampling with individuals collected in May. As such, the short‐term (10‐day) difference in environmental conditions was largest for temperature (Figure 1c). In total, there was 201 (6.6%) 100 bp methylated regions identified as significantly differentially methylated in at least one of the three comparison with the other sites. Of these, only two DMRs were differentially methylated in all three population comparisons against LF. Comparing LF with the low salinity site, VB identified 130 DMRs while contrasts with the mid‐salinity and high salinity sites (SL and LC) contained 38 and 60 DMRs, respectively. Across all regions of gene bodies (2 kb upstream to 2 kb downstream), the most significant (lowest *q*‐value) hypomethylated DMRs in the LF population were associated with genes coding for the gametocyte surface protein P230, a Protein‐L‐isoaspartate(D‐aspartate) O‐methyltransferase enzyme, a histone acetyltransferase KAT2B‐like gene, the enzyme ketohexokinase‐like (Table 1). The hypermethylated DMRs in the LF population were associated with gene bodies of a germinal center associated nuclear protein, a palmitoyltransferase ZDHHC17‐like gene, a protogenin B‐like gene, and a DNA‐directed DNA polymerase (Table 1; Appendix S2). GO enrichment identified significant enrichment between LF and VB of the broad category *extracellular region*.

The Sister Lake site has the second lowest annual mean salinity spending approximately 71.4% of the year below 15 psu and 7.2% of the year below 3.5 psu (Figure 1). There were a total of 111 (8.5%) DMRs between Sister Lake and any of the other three sites. Of these, only six DMRs were differentially methylated in all three population comparisons against SL. Pairwise differences in methylation identified 54 DMRs when compared with the high salinity site—LC, 38 DMRs when compared with the mid‐salinity site—LF, and 48 DMRs when compared with the low salinity site—VB. Hypomethylation of gene bodies in the SL population was associated with a uncharacterized lncRNA, an insulin‐like peptide receptor, and a gene coding for the centromere‐associated protein E. Hypermethylated DMRs in the SLpopulation were associated with a dual specificity protein phosphatase 22, a dnaJ homolog subfamily C member 16‐like gene, and condensin complex subunit 1. GO enrichment also identified significant enrichment between SL and VB of the broad category *extracellular region*.

Vermillion Bay has the lowest annual mean salinity of all sites with 99.8% of the year below 15 psu, 57% of the year below 3.5 psu, and had a 10 day mean salinity of 2.9 prior to sampling (Figure 1). Vermillion Bay was also identified as having the highest number of DMRs when compared with all other sites with 253 (6.95%) of the 100 bp regions identified as significantly differentially methylated in at least one of the 3 pairwise comparisons. Pairwise differences in methylation identified 103 DMRs when compared with the high salinity site—LC, 130 DMRs when compared with the mid‐salinity site—LF, and 48 DMRs when compared with SL. Hypomethylation of gene bodies in the VB population was associated with the gametocyte surface protein P230, a cytosolic carboxypeptidase 1‐like protein, and an uncharacterized long noncoding RNA. Hypermethylated DMRs in the VB populations included genes associated with a germinal center associated nuclear protein‐like, a cytoplasmic dynein 1 heavy chain 1‐like isoform X1, and a palmitoyltransferase ZDHHC17‐like isoform X1. GO enrichment also identified significant enrichment between VB and both the LF and SL populations of the same broad category *extracellular region*. Genes associated with this ontology in both comparisons included regulatory genes such as WNT‐5b, neurogenic locus notch homolog protein 4‐like, and a phosphatidylinositol‐glycan‐specific phospholipase D‐like gene (Appendix S2).

### 3.6 Differential methylation between populations identified in conStruct

The final comparison of differences in methylation between populations used the results obtained from the conStruct analysis identified significant genomic structure at *K* = 3, with nearly equal support for *K* = 2. After assessment of the structure plot in Figure 2, we focused on exploring differences in methylation between a *K* = 2 model wherein the individuals sampled from Lake Calcasieu and Sister Lake were considered to be population 1 while the individuals sampled at Lake Fortuna and Vermilion Bay were considered to be population 2. This approach was chosen to test if the genomic population structure might explain the distribution of methylation between populations. Hierarchical clustering comparing population 1 with population 2 identified clustering based on population with only minimal overlap of individuals (Figure 5a). Differences in the 10‐day mean salinity and temperatures between the two population groups showed that population 1 was on average 4 psu higher than population 2, but population 2 was 7°C warmer than population 1. Global methylation patterns between these two meta‐populations using a pairwise assessment of differential methylation identified 282 DMRs. The top DMRs within gene bodies were hypomethylated in population 2 (LF + VB) when compared with population 1 (LC + SL) and were associated with genes coding for a histone acetyltransferase KAT6A‐like gene, a anoctamin‐7‐like gene, a ATP‐binding cassette sub‐family A member 3‐like gene, and a glutathione synthetase‐like gene. The top hypermethylated DMRs were associated with a V‐type proton ATPase subunit, a protogenin B‐like gene, a sodium/hydrogen exchanger 9B2 gene, and dnaJ homolog subfamily C member 1‐like gene (Table 2). Gene ontology enrichment between these two populations revealed significant enrichment among hypermethylated genes associated with the ontology *transferase activity, transferring glycosyl groups*. Among the genes that were hypomethylated within the transferase activity was a glycylpeptide N‐tetradecanoyltransferase 2‐like gene that had a mean methylation of 7.7% methylation in population 2 and a mean methylation of 27.5% in population 1. This relationship was also true for the ATP‐binding cassette sub‐family A member 3‐like gene and an uncharacterized protein.

**Table 2:** Significantly differentially methylated regions (DMRs) identified from the *K* = 2 population comparisons

Gene name	Gene ID	Chromosome	Start	Methylation difference (%)	*q*‐value
LOW QUALITY PROTEIN: anoctamin−7‐like	gene17601	NC_035784.1	13,100,700	−28.11	5.67E−12
Histone acetyltransferase KAT6A‐like	gene10109	NC_035782.1	36,268,900	−26.38	1.11E−06
Ketohexokinase‐like	gene19389	NC_035784.1	38,858,800	−23.98	1.24E−12
ATP‐binding cassette sub‐family A member 3‐like	gene29286	NC_035787.1	12,555,600	−20.64	2.16E−40
Glutathione synthetase‐like	gene28446	NC_035786.1	55,258,701	−17.58	2.30E−09
Protogenin B‐like	gene38444	NC_035789.1	8,868,800	19.50	1.03E−19
DnaJ homolog subfamily C member 1‐like	gene36200	NC_035788.1	67,912,000	20.36	1.91E−11
V‐type proton ATPase subunit E‐like	gene24127	NC_035785.1	23,166,100	22.66	1.90E−33
LOW QUALITY PROTEIN: sodium/hydrogen exchanger 9B2‐like	gene5344	NC_035781.1	20,161,600	29.75	1.99E−15
Transient receptor potential cation channel subfamily M member 2‐like isoform X2	gene5144	NC_035781.1	16,979,600	47.30	2.85E−35

Note

A full list of DMRs is available in Appendix S2.

**Figure 6:**
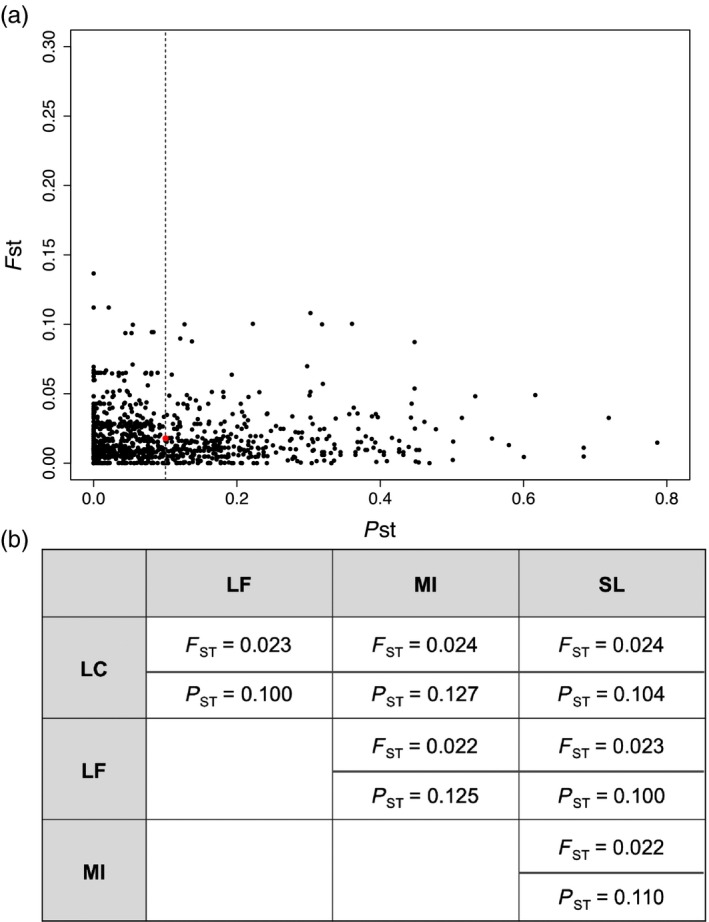
(a) Per locus *P*_ST_ plotted against *F*_ST_ for loci shared between the two datasets. The red spot identifies the intersection of the observed mean *F*_ST_ and mean *P*_ST_ for all loci. (b) Pairwise estimates of *F*_ST_ and *P*_ST_ for all loci

Finally, assessing variation between genomic variation (*F*_ST_) and our estimate of epigenetic variation (*P*_ST_) identified a mean *P*_ST_ of 0.10 and a mean *F*_ST_ of 0.023. This suggests that there is almost 5 times greater variation between populations in methylation than in variation of *F*_ST_ (Figure 6a). This separation in *P*_ST_ and *F*_ST_ was also seen in each of the pairwise comparisons between populations (Figure 6b). This analysis did not reveal any correlation between regions with elevated *P*_ST_ and *F*_ST._ Finally, analyzing this combination of genomic and epigenetic data allowed us to identify differential methylation across multiple genomic features in light of the genomic evidence for population structure.

## 4 DISCUSSION

There is an emerging interest in understanding the role DNA methylation plays in population responses to heterogeneous environments (Hofmann, 2017; Hu & Barrett, 2017; Metzger & Schulte, 2016). Recent physiological evidence suggests there is local adaptation between populations of *C. virginica* distributed across the northern Gulf of Mexico (GOM) (Leonhardt et al., 2017). Therefore, this study was designed to assess genetic and epigenetic variation between oysters collected from four coastal Louisiana sites spanning the range of salinity conditions inhabited by oysters in the GOM. By employing a combination of RADseq and comparative epigenomic analysis of oysters from these four sites, we were able to show significant population structure for genetic sequences, but much greater divergence in population specific methylation. This suggests that DNA methylation may play a greater role than sequence divergence in generating the previously observed phenotypic differences among *C. virginica* populations. While there are potential limitations to these data owing to the temporal distribution of collections and variation in prior exposure, these data provide important insight into epigenetic variation across known environmental gradients that may be contributing to the establishment of acclimatization and local adaptation among populations of *C. virginica* in this region.

### 4.1 Genetic population structure

A surprising result from our analysis was the observed genetic similarities did not follow a geographic pattern, with the greatest similarities between Lake Fortuna and Vermillion Bay and between Lake Calcasieu and Sister Lake (Figure 2). We had originally hypothesized that Lake Fortuna would be the most divergent population as the Mississippi river presents a significant barrier to larval dispersal along coastal Louisiana. Our findings may reflect the effects of human management. In Louisiana, juvenile (seed) oysters are routinely moved from public seed grounds to private oyster leases. Public seed grounds have historically included Lake Calcasieu, Lake Fortuna, and Vermillion Bay, and there are private leases within or near Lake Fortuna, Vermillion Bay, and Sister Lake. In addition, hatchery produced larvae or juveniles have been outplanted at several of these locations by the Louisiana Department of Wildlife and Fisheries (Leonhardt, 2013). The influence of these oyster transplants and translocations may also explain the results from the conStruct analysis which suggests that the observed population structure is not driven by an isolation‐by‐distance effect. The conStruct analysis also identified at least two distinct populations at each site, possibly reflecting the presence of both “native” and translocated or hatchery reared genotypes. Previous studies have observed similar patterns that suggest RADseq approaches are valuable tools for identifying the influence of management activities on population structure (Bernatchez et al., 2019; Silliman, 2019).

### 4.2 Global DNA methylation patterns in C. virginica

The methylome sequencing approach used in this study revealed that approximately 14% of the *C. virginica* genome is methylated. This level of methylation is comparable to previous studies that report 15% of the *C. gigas* genome as being methylated (Gavery & Roberts, 2013; Olson & Roberts, 2014). The distribution of methylated regions across genomic features was also very similar to *C. gigas,* with a slightly higher percentage of regions within promoter regions being identified as methylated in *C. virginica* (Gavery & Roberts, 2013). The observed overlap in frequency and distribution of methylation within gill tissue between these two closely related species suggests a conserved role for DNA methylation within oyster genomes. Furthermore, the population differences observed here are a snap‐shot of methylation states that are shaped by the environmental conditions at the time of collection. The presence of shared methylation states between individuals within a site strengthens the case for exploring potential intergenerational inheritance of these methylation patterns as was recently demonstrated in *C. gigas* following parental exposure to the herbicide diuron (Rondon et al., 2017).

The range of population specific differentially methylated regions (38–282 regions) was consistently relegated to less than 10% of all measured regions (6.7%–8.6%), suggesting that the majority of methylation within the *C. virginica* genome is fixed and only a small portion is plastic. This is consistent with the concept that the major function of epigenomic modification is to control cellular differentiation and that environmentally induced de novo modifications are primarily important for maximizing phenotypic plasticity at both the cellular and organismal level (Eirin‐Lopez & Putnam, 2018). Therefore, focusing on the genomic regions that are associated with DMRs between the same tissue types from individuals collected at the same life‐history stages will provide further evidence for which genes are important for maximizing genome—phenome diversity across a range of environmental conditions.

Among all pairwise assessment of differential methylation, gene ontology enrichment was only identified between comparisons of the low salinity site Vermillion Bay and both Lake Fortuna and Sister Lake. In both cases, the enrichment was among the broad category *extracellular region*. Genes associated with this ontology were hypermethylated in Vermilion Bay and may reflect differences in developmental stages between individuals at these sites. One caveat regarding these data is that the collections presented here was measured on individuals that had significant variation in size; however, growth rate in *C.virginica* is dependent on environmental conditions; therefore, attributing age based on size is not a dependable method when comparing oysters from estuaries known to differ in salinity. However, presence or absence of gonads as was seen among individuals from Lake Fortuna suggests differences in reproductive status may have an influence on DNA methylation. Evidence for this can be seen in the pairwise assessments focused on the Lake Fortuna population that identified significant hypermethylation of genes associated with developmental processes. Specifically, the hypermethylation of protogenin B and germinal center associated nuclear protein may reflect seasonal changes in DNA methylation that could be associated with reproductive timing. Future studies that account for these components will be crucial in validating the results presented here as samples varied in both time of collection and age of individuals.

### 4.3 DNA methylation and population structure

Integrating the conStruct structure results from the RADseq analysis provided an important insight into the influence of genotype on DNA methylation. Specifically, adjusting the differential methylation comparisons to reflect a population structure of *K* = 2 identified 282 DMRs that showed enrichment for genes associated with transferring glycosyl groups. Specifically, enrichment was seen among hypomethylated genes coding for a gene with enzyme annotation of glycosyltransferase and the gene coding for a glutathione synthetase‐like protein. These enzymes are important for the biosynthesis of many carbohydrates and detoxification that have been shown to be differentially regulated, at the transcript level, in response to stress in other *Crassostrea species* (Müller et al., 2018; Tanguy, Boutet, & Moraga, 2005). The hypermethylation of ketohexokinase, an enzyme involved in metabolism, suggests that differences in food availability between the estuaries may influence these methylation patterns. The hypermethylation of ion channels such as sodium/hydrogen exchanger 9B2 and the transient receptor potential cation channel suggests that the salinity differences between the two population groups may also influence differential methylation (Jones et al., 2019). Finally, the hypomethylated DMR located within the gene body of the ATP‐binding cassette sub‐family A member 3‐like gene was found to be one of three regions that had less than 10% methylation in population 2. This gene is putatively involved in shell formation, and the transition from 7.5% methylation to 36.2% methylation may reflect physiologically significant changes in methylation (Feng, Li, Yu, Zhao, & Kong, 2015).

The relationship between genetic and epigenetic population structure suggests some influence of genetic structure on methylation state (see Figure 5). Despite this influence, the greater differences among populations in methylation suggests there is potentially both an environmental and developmental influence on epigenetic methylation status. While the RADseq analysis suggests that genetic structure may also be influenced by management related activities, the presence of a strong differential methylation of ion transporters and genes involved in stress responses provides evidence that populations have unique methylome profiles that may directly regulate the plasticity of these genes in response to variation in the environment.

## 5 CONCLUSION

Our analyses identified differential methylation among genes known to exhibit high plasticity in response to environmental conditions (sodium/hydrogen exchanger, glutathione synthetase), to developmental timing (protogenin B), and DNA damage repair (dnaJ homologs). The variation in methylation around these genomic features potentially reflects both differences in timing of collection and differences in the environments the individuals were collected from. The DMRs identified here serve to highlight the diversity of methylation in the Eastern oyster and provide further evidence of the dynamic nature of CpG methylation in response to both environmental conditions and phenology. In addition, global methylation patterns revealed that relationships identified in *C. gigas* holds true for *C. virginica* and therefore supports the role of CpG methylation in phenotype environmental interactions for the genus *Crassostrea*. Assessing methylation patterns between populations identified using RADseq analysis also highlighted the effects of genomic structure on DNA methylation. Finally, our RADseq analysis also identified unexpected population structure potentially arising from management practices that may act counter to local adaptations to salinity within this coastal system. These results reinforce the need to combine genomic and epigenomic data when seeking to understand divergent population responses to heterogeneous environments.

## CONFLICT OF INTEREST

None declared.

## Data Availability

Data for this study will be made publicly available through the NCBI SRA database under accession PRJNA488288. STACKS code is available in Appendix S1, and all R code associated with this analysis is archived on Zenodo at https://doi.org/10.5281/zenodo.3551408.
