# Supplementary material for: Population epigenetic divergence exceeds genetic divergence in the Eastern oyster Crassostrea virginica in the Northern Gulf of Mexico
Source: Evol Appl. 2020 Jan 7;13(5):945–59. doi: 10.1111/eva.12912 (PMC7232765; doi:10.1111/eva.12912)
Supplement: Supplementary file 1 [file EVA-13-945-s001.docx]

**EpiGBS methods**

based on:

van Gurp et. al. epiGBS: reference-free reduced representation bisulfite sequencing

*Nature Methods* 13, 322–324 (2016) doi:10.1038/nmeth.3763.

**Starting material :** 400 ng of gDNA (260/280 > 1.8; 260/230 >1.5)

**Bisulfite conversion kit:**

EZ DNA Methylation-Lightning Kit (Zymo Research)

**PCR Cleanup kit:**

Qiaquick PCR cleanup (Qiagen; 28104)

**Library quality:**

high-sensitivity DNA chip on a 2100 Bioanalyzer system or tapestation

| Y-yoke_adapter_1a | 5’-/5Phos/AXAXTXTTTXXXTAXAXGAXGXTXTTXXGATC-3’ |
| --- | --- |
| Y-yoke_adapter_1b | 5'-TAGATXGGAAGAGXAXAXGTXTGAAXTXXAGTXAX-3' |
| Y-yoke_adapter_2a | 5'-/5Phos/AXAXTXTTTXXXTAXAXGAXGXTXTTXXGATCTGXAT-3' |
| Y-yoke_adapter_2b | 5’-GATXGGAAGAGXAXAXGTXTGAAXTXXAGTXAX-3’ |
| Illumina PE-PCR primer 1 | 5’-AATGATACGGCGACCACCGAGATCTACACTCTTTCCCTACACGACGCTCTTCCGATCT-3’ |
| Illumina PE-PCR primer 2 | 5’-CAAGCAGAAGACGGCATACGAGATCGGTCTCGGCATTCCTGCTGAACCGCTCTTCCGATCT­­­­­-3’ |

* X = 5-methyl-cytosine

**Reagents:**

- FastDigest buffer
- BSA (NEB; B9000S)
- AseI and NsiI (NEB-R0127L and NEB-R0526L
- T4 DNA ligase (NEB; MO202M/L)
- T4 DNA ligase buffer
- Agencourt AMPure XP beads (Beckman Coulter; A63880)
- 10mM 5-methylcytosine dNTP mix (Zymo Research; D1030)
- NEBuffer 2
- rATP (NEB)
- DNA polumerase I (NEB; M0209S)
- KAPA HiFi HotStart Uracil+ ReadyMix (Kapa Biosystems)

**Methods:**

1. Restriction digest

- Digest 500 ng of gDNA overnight (17hr) at 37 °C in a volume of 40 μL containing 1× FastDigest buffer, 125 μg of BSA (NEB; B9000S) and 40 units of AseI and NsiI (NEB-R0127L and NEB-R0526L).

1. Adapter Ligation (60 μl total volume)
   1. 40 μl digested gDNA
   2. 1x T4 DNA ligase buffer
   3. 4,000 units of T4 DNA ligase (NEB; MO202M/L)
   4. 2,400 pg of both forward and reverse barcoded adapters
   5. ligate for 3hr at 22 °C and overnight at 4 °C with no inactivation afterward
2. Cleanup and Size Select
   1. Use Qiaquick PCR cleanup (Qiagen; 28104) to reduce volume to 60 μl
   2. Size select with 0.8x Agencourt AMPure XP beads (Beckman Coulter; A63880)
   3. Elute size select into 24 μl
3. Nick translation
   1. Combine 18 μl purified library with 2.5 μl 10mM 5-methylcytosine dNTP mix (Zymo Research; D1030), 1x NEBuffer 2, and 7.5 units of DNA polumerase I (NEB; M0209S)
   2. Incubate reaction for 1hr at 15 C
4. GBS PCR library quality check
   1. 1 μl ssDNA template
   2. 5 μl KAPA HiFi HotStart Uracil+ ReadyMix (Kapa Biosystems)
   3. 3 pmol of each Illumina PE PCR primer
      1. 95°C for 3min
      2. 18 cycles of:
         1. 98°C for 10s
         2. 65°C for 15 s
         3. 72°C for 15s
      3. 72°C for 5 min
   4. Assess the quality of the PCR libraries by analyzing 1 μL of the PCR product on a high-sensitivity DNA chip on a 2100 Bioanalyzer system or tapestation
5. Bisulfite treatment and purification
   1. Input: 20 μl of nick-translated libraries
   2. Kit: EZ DNA Methylation-Lightning Kit (Zymo Research)
      1. 98°C for 8 min
      2. 54°C for 1 hr
      3. 4°C for up to 20 hr
6. Library amplification (Run 4 10- μl reactions for each library!
   1. 1 μl ssDNA template
   2. 5 μl KAPA HiFi HotStart Uracil+ ReadyMix (Kapa Biosystems)
   3. 3 pmol of each Illumina PE PCR primer
      1. 95°C for 3min
      2. 15 cycles of:
         1. 98°C for 10s
         2. 65°C for 15 s
         3. 72°C for 15s
      3. 72°C for 5 min
   4. Assess the quality of the PCR libraries by analyzing 1 μL of the PCR product on a high-sensitivity DNA chip on a 2100 Bioanalyzer system
   5. Good libraries were between 150-400 bp with no adapter dimers
   6. Typically epiGBS PCR reactions of 16 cycles of a non-pooled sample yielded 3-12 ng/ul of product

**STACKS code**

#!/bin/bash

#PBS -q checkpt

#PBS -l nodes=1:ppn=16

#PBS -l walltime=72:00:00

#PBS -o ~/FastqFiles_MS2_044_OysterRADseq

#PBS -j oe

#PBS -N out_demultiplex

module load gcc/6.4.0

/home/usr/stacks-2.3d/process_radtags \

-P -p ~/FastqFiles_MS2_044_OysterRADseq/raw \

-b ~/FastqFiles_MS2_044_OysterRADseq/demulti --inline_inline \

-o /work/c/FastqFiles_MS2_044_OysterRADseq/radtag_proccess_out \

-i gzfastq \

-c -q -r -t 140 -w 0.15 -s 10 \

--renz_1 xbaI \

--renz_2 ecoRI \

--adapter_mm 2 \

--adapter_1 AGATCGGAAGAGCACACGTCTGAACTCCAGTCAC \

--adapter_2 AGATCGGAAGAGCGTCGTGTAGGGAAAGAGTGT

OK, now using bwa for sequence alignment (using BWA because that is what STACKS recommends)

#!/bin/bash

#PBS -A hpc_18_4

#PBS -q checkpt

#PBS -l nodes=1:ppn=16

#PBS -l walltime=72:00:00

#PBS -o ~/FastqFiles_MS2_044_OysterRADseq

#PBS -j oe

#PBS -N out_bwa

module load gcc/6.4.0

module load samtools/1.9/INTEL-18.0.0

cd ~/FastqFiles_MS2_044_OysterRADseq/

for fname in ~/FastqFiles_MS2_044_OysterRADseq/radtag_proccess_out/samples2/*.1.fq.gz

do

base=${fname%.1.*}

/home/usr/bwa/bwa mem -t 16 ~/CV_genome/CV_genome "${base}.1.fq.gz" "${base}.2.fq.gz" >"$destdir/${base}.sam" ;

done

###The output sam files will now need to be sorted before being used in STACKS

#!/bin/bash

#PBS -A hpc_18_4

#PBS -q checkpt

#PBS -l nodes=1:ppn=16

#PBS -l walltime=72:00:00

#PBS -o ~/FastqFiles_MS2_044_OysterRADseq

#PBS -j oe

#PBS -M user

#PBS -N out_samtools

module load gcc/6.4.0

module load samtools/1.9/INTEL-18.0.0

cd ~/FastqFiles_MS2_044_OysterRADseq/radtag_proccess_out/samples2/

for fname in ~/FastqFiles_MS2_044_OysterRADseq/radtag_proccess_out/samples2/*.sam

do

base=${fname%.sam}

samtools view -b "${base}.sam"|

samtools sort --threads 16 > "$destdir/${base}.bam" ;

done

Sorted files are now ready for stacks ref_map.pl pipeline.

#!/bin/bash

#PBS -A hpc_18_4

#PBS -q checkpt

#PBS -l nodes=1:ppn=16

#PBS -l walltime=72:00:00

#PBS -o ~/FastqFiles_MS2_044_OysterRADseq

#PBS -j oe

#PBS -M user

#PBS -N out_samtools

module load gcc/6.4.0

module load samtools/1.9/INTEL-18.0.0

cd ~/FastqFiles_MS2_044_OysterRADseq/radtag_proccess_out/samples2/

perl stacks-2.41/ref_map.pl \

-T 16 \

--popmap popmap.txt \

--rm-pcr-duplicates \

-o stacks/STRUCTURE \

-X "populations: -p 4 --smooth --hwe -r 0.65 --min_maf 0.05 --bootstrap --bootstrap-reps 1000000 -t 16 --structure --genepop --write_single_snp --vcf" \

--samples bam/

This provides both pair-wise Fst estimates in addition to a structure file that is ready for construct analysis in R.
